# Factors associated with adherence to antihypertensive agents in the older adult

**DOI:** 10.15649/cuidarte.3474

**Published:** 2024-05-28

**Authors:** Jack Roberto Silva Fhon, María del Pilar Gómez-Luján, Gideany Maiara Caetano, Giovanna Sara Cáceda-Ñazco, Alexandre Pereira dos Santos-Neto, Zoila Esperanza Leitón-Espinoza

**Affiliations:** 1 University of São Paulo, São Paulo, Brazil. E-mail: betofhon@usp.br Universidade de São Paulo University of São Paulo São Paulo Brazil betofhon@usp.br; 2 National University of Trujillo, Trujillo, Peru. E-mail: mpgomezl@unitru.edu.pe Universidad Nacional de Trujillo National University of Trujillo Trujillo Peru mpgomezl@unitru.edu.pe; 3 University of São Paulo, São Paulo, Brazil. E-mail: gideany.caetano@usp.br Universidade de São Paulo University of São Paulo São Paulo Brazil gideany.caetano@usp.br; 4 National University of Trujillo, Trujillo, Peru. E-mail: gcaceda@unitru.edu.pe Universidad Nacional de Trujillo National University of Trujillo Trujillo Peru gcaceda@unitru.edu.pe; 5 University of São Paulo, São Paulo, Brazil. E-mail: alexandre.neto@usp.br Universidade de São Paulo University of São Paulo São Paulo Brazil alexandre.neto@usp.br; 6 National University of Trujillo, Trujillo, Peru. E-mail: zoilaeleiton@gmail.com Universidad Nacional de Trujillo National University of Trujillo Trujillo Peru zoilaeleiton@gmail.com

**Keywords:** Hypertension, Geriatric Nursing, Treatment Adherence and Compliance, Aged, Hipertensión, Enfermería Geriátrica, Cumplimiento y Adherencia al Tratamiento, Anciano, Hipertensao, Enfermagem Geriátrica, Cooperagao e Adesao ao Tratamento, Idoso

## Abstract

**Introduction:**

With aging, there is an increased risk of suffering from different chronic diseases, including high blood pressure. Hypertension management must be carried out by health professionals, whether or not treatment involves medication. By controlling drug treatment, especially adherence, serious health problems for older people can be avoided.

**Objective:**

To determine the factors associated with adherence to arterial hypertension treatment in older adults who live at home.

**Materials and Methods:**

A quantitative and cross-sectional study was conducted in La Libertad Region, Peru, with 342 older adults living at home. For data collection, a sociodemographic profile form, anthropometric measurements, blood pressure measurements, the Mini-mental State Examination (MMSE) test, the Geriatric Depression Scale (GDS), and the Morisky Green Levine (MGL) adherence scale were used. In addition, descriptive and analytical statistics were used.

**Result:**

57.60% of the participants did not adhere to the pharmacological treatment, and, in most of the sociodemographic variables examined, they did not adhere to pharmacological treatment in most cases. Likewise, a relationship between retirement in older adults and the MGL adherence scale score was identified. The study showed evidence linking treatment adherence and age (p=0.01), retirement status (p=0.05), and history of stroke (p=0.004).

**Discussion:**

Treatment adherence depends on sociodemographic and health factors for disease control and a healthy lifestyle.

**Conclusion:**

Older adults and their caregivers need guidance and education to improve adherence to pharmacological treatments.

## Resumen

**Introducción:**

Con el envejecimiento, aumenta el riesgo de padecer distintas enfermedades crónicas, incluida la hipertensión arterial. El manejo de la hipertensión debe realizarlo profesionales de la salud, sea que el tratamiento incluya o no medicamentos. El control del tratamiento farmacológico, sobre todo la adherencia, evitará graves consecuencias para la salud de las personas mayores.

**Objetivo:**

Determinar los factores asociados a la adherencia al tratamiento de la hipertensión arterial en adultos mayores que viven en su hogar.

**Materiales y Métodos:**

Estudio cuantitativo y transversal realizado en la región La Libertad, Perú, con 342 adultos mayores que viven en sus hogares. Para recoger la información, se utilizaron los instrumentos de perfil sociodemográfico, medidas antropométricas, medición de la tensión arterial, el miniexamen del estado mental, la escala de depresión geriátrica y la escala de adherencia de Morisky,Green y Levine (MGL). Además, se utilizaron estadísticas descriptivas y analíticas.

**Resultados:**

Se identificó que el 57,60% de los participantes no cumplían el tratamiento farmacológico en la mayoría de las variables sociodemográficas estudiadas. Asimismo, se identificó una relación entre la jubilación en adultos mayores y el puntaje en la escala MGL. El estudio mostró evidencias de una asociación entre la adherencia al tratamiento y la edad (p=0,01), estar jubilado (p=0,05) y tener antecedentes de accidente cerebrovascular (p=0,004).

**Discusión:**

La adherencia al tratamiento depende de factores sociodemográficos y de salud para el control de la enfermedad, además de un estilo de vida saludable.

**Conclusión:**

Los adultos mayores y sus cuidadores necesitan orientación y educación para mejorar la adherencia al tratamiento farmacológico.

## Resumo

**Introdujo:**

Com o processo de envelhecimento, aumenta o risco de sofrer de diversas doengas crónicas, incluindo hipertensao arterial. Esse manejo deve ser realizado por profissionais de saúde com tratamento com e sem medicamentos. O tratamento medicamentoso deve ser controlado, principalmente na adesao ao mesmo, pois evitará consequencias graves a saúde do idoso.

**Objetivo:**

determinar os fatores associados a adesao ao tratamento da hipertensao arterial em idosos que residem no domicílio.

**Materiais e Métodos:**

Estudo quantitativo e transversal realizado na regiao de La Libertad, Peru, com 342 idosos que moram no domicilio. Para a coleta de informales foram utilizados os instrumentos de perfil sociodemográfico, medidas antropométricas, medida de pressao arterial, miniexame do estado mental, escala de depressao geriátrica e teste de Morisky- Green. Além disso, foram utilizadas estatísticas descritivas e analíticas.

**Resultado:**

Identificou-se que 57,60% dos participantes nao aderiram ao tratamento farmacológico com a maioria das variáveis sociodemográficas estudadas. Da mesma forma, foi identificada relagao com a aposentadoria em idosos e o teste de Morisky-Green. O estudo mostrou evidencias de associagao entre idade (p=0,01), estar aposentado (p=0,05) e ter sofrido acidente vascular cerebral (p=0,004) com a adesao ao tratamento.

**Discussao:**

a adesao ao tratamento depende de fatores sociodemográficos e de saúde para o controle da doenga, além de um estilo de vida saudável.

**Conclusao:**

Os idosos e seus cuidadores necessitam de orientado e educagao para melhorar a adesao ao tratamento farmacológico.

## Introduction

The world's nations are witnessing an increase in the number and proportion of older people, with an unusual rise in recent decades. Consequently, as of the outset of 2020, global reports indicated a figure of over one billion older adults worldwide, constituting approximately 13.5% of the world population^1^.

Along with the demographic change and the increase in the older adult population, there is a gradual uptick in non-communicable chronic diseases. Among these, high blood pressure (HBP) stands out, affecting more than 1,500 million people globally and resulting in 9,4 million deaths annually worldwide^2^.

The prevalence of HBP ranges between 30 and 45% in the general population and between 70 and 77% in the older adult population, a major concern for international health authorities^3^.

Hypertension is an alteration in arterial blood pressure in which the systolic pressure is equal to or greater than 140 mm/Hg, or the diastolic pressure is equal to or greater than 90 mm/Hg. It can be influenced by intrinsic factors such as genetics and extrinsic factors, such as changes in diet, unhealthy lifestyle, obesity, sodium and potassium intake, air pollution, lead, and psychosocial stress, among others^4^.

Although HBP can be controlled through pharmacological treatment, achieving effective stability remains challenging for health professionals due to reasons attributable to either physicians or patients. Among these reasons, inadequate therapeutic management, satisfaction with moderate reductions in blood pressure linked to patients' lack of commitment, a silent progression of the disease, medication cessation, and poor adherence to the treatment are notable^5^.

Patient adherence to treatment can be qualitatively reported as adherent or non-adherent. It is defined as the number of pills taken during a specific period compared to the number of pills prescribed by a doctor during the same period^6^. In addition, adherence to treatment is considered a complex behavior formed by an internal structure and dynamics, made up of personal, relational, and behavioral components: personal, related to oneself; relational, in which health professionals are involved; and behavioral, aimed at achieving a beneficial health result^7^.

Adherence to treatment can be measured in different ways. Direct methods include measuring the concentration of drug or metabolite in blood and urine, detecting the presence of a biomarker supplied with the drug, and observing patients' drug-taking behavior. Indirect methods include the use of various questionnaires^8^.

In a study conducted in the United States, 81.5% of people reported suffering from HBP, 74.9% were being treated, and 52.5% had their HBP controlled, results that can be attributed to low adherence to treatment and have serious repercussions on health^9^. On the other hand, a Chinese study with 488 participants found that only 27.46% had good adherence to treatment and that sex, type of housing, occupation, and duration of treatment contributed to this percentage of adherence^10^.

Poor adherence to treatment is an important factor limiting the benefits of antihypertensive therapy in all age groups, but especially in older people^11^. Low adherence has been associated with several problems, including high incidence and mortality from strokes, higher hospitalization rates, and high healthcare costs^12^.

Patients' lack of knowledge, beliefs, and myths about HBP negatively impact adherence. In this sense, this study will provide information on how older people manage HBP and how health professionals can devise strategies to improve adherence. Therefore, this study aimed to determine the factors associated with adherence to HBP treatment among older adults living at home.

## Materials and Methods

A quantitative, observational, and cross-sectional study was conducted in northern Peru, specifically in the La Libertad region. This region encompasses several provinces, including Trujillo, Chepén, Santiago de Chuco, Ascope, Otuzco, Bolivar, Julcan, Pacasmayo, Sánchez Carrión, Pataz, Virú, and Gran Chimú. Trujillo is the province with the largest older population^13^; therefore, 11 of its districts were considered. The studio data is stored in Mendeley Data^14^.

The study encompassed a population of 131,465 older adults. A 5% significance level and a sampling error of ±3 were used for sample size calculation, and a sample of 1,058 older individuals was established. However, to mitigate potential loss of information or data entry errors, an additional 5%, totaling 56 more surveys, were conducted, resulting in a sample size of 1,110 older adults. From this sample, a subsample of 342 individuals who reported suffering from HBP was obtained.

To survey each district, a zoning division was established, specifying the number of blocks and households within each block. It was determined that four surveys would be conducted in each block, ensuring comprehensive coverage across all zones. Once the household was located, individuals to be interviewed were selected using Leslie Kish's table to ensure everyone had an equal opportunity to participate^15^. Data were collected by nurses, teachers, and nursing students with prior training.

Participants were required to meet the following inclusion criteria: age 60 or older, either male or female, physically and mentally fit or not, and suffering from HBP. Participants who did not receive on more than two occasions the interviewers were excluded from the study.

The interviews lasted around 40 minutes, took place at the participant's home, and were arranged in advance to accommodate participants' scheduling and availability. The following instruments were used to collect the information:

*Demographic profile form:* It gathered information regarding sex (male and female), age (years), marital status (with and without a partner), level of education (without education, primary education, secondary and higher education), wage (Peruvian soles), retiree (yes or no), and living alone (yes or no).

*Life habits questionnaire:* In the case of smoking and drinking alcoholic beverages, participants were asked if they had consumed any in the last three months.

*Anthropometric measurements:* Weight, height, mid-upper arm circumference (MUAC), and calf circumference (CC) were measured. Height and weight were measured following the Centers for Disease Control and Prevention (CDC) recommendations on measuring height and weight at home^16^.

For MUAC measurement, participants were asked to bend the arm at a right angle at the elbow, with the palm facing upward. The distance between the acromion of the scapula (the protruding bony surface of the shoulder’s upper part) and the olecranon (elbow’s bony tip) was then measured, and the midpoint between the two was marked. Next, participants were asked to let their arm hang relaxed at their side, and the tape measure was placed well-adjusted at the midpoint of the arm, avoiding pinching and excessive pressure^17^. Measurements were scored as follows: MUAC < 21cm = 0.0 points; MUAC from 21 to 22cm = 0.5 point, and MUAC > 22cm = 1.0 points^18^.

For CC measurement, participants were asked to sit with their leg dangling, the tape measure was placed around the calf at its widest point, and the measurement was rounded to the nearest millimeter. For correct measurement, the tape measure was at a right angle to the calf length^17^. Measurements were scored as follows: CC < 31cm = 0 points, CC >31cm = 1^18^.

*Body Mass Index (BMI):* Using weight and height measurements, the BMI was calculated using the following formula: weight (kg) / [height (m)]2. The results were interpreted as follows: <18.4 = malnutrition, 18.5 to 21.9 = underweight, 22 to 26.9 = normal weight, 27 to 29.9 = overweight, and >30 = obesity^19^.

*Blood pressure measurement:* ®Riester blood pressure cuff for adults was used to measure blood pressure, and the procedure described in WHO STEPS Surveillance Manual: the WHO STEPwise method for blood pressure surveillance^20^ was followed. Blood pressure was categorized as follows: normal (<120 - <80 mm/Hg); pre-hypertension (120-139 to 80-89mm/Hg); stage 1 hypertension (140 159 to 90-99 mm/Hg), and stage 2 hypertension (>160 to >100)^21^.

*Self-reported illnesses:* The participants were asked about any illnesses or health problems. If they responded affirmatively, they were prompted to specify the nature of those illnesses. Subsequently, the identified diseases were named and coded according to the Major Diagnostic Categories (MDC) and Diagnosis Related Groups (AP-GRD v 25.0)^22^.

*Mini-mental State Examination (MMSE):* It assesses orientation, registration, attention and calculation, recall, language, and constructive praxis. The MMSE has been validated and widely used in both clinical practice and research. In Latin America, MMSE was adapted and validated in Chile for the PAHO Health, Well-being and Aging (SABE) survey^23^.

A score cut-off of 12/13 was established to distinguish individuals with cognitive impairment: those with 13 or more points were classified as normal, whereas those with 12 or fewer points were suggestive of cognitive impairment^23^.

*Mini Nutritional Assessment Short Form (MNA-SF):* This form allows the assessment of the risk of malnutrition among individuals aged 60 and over. It includes 18 questions addressing anthropometric measurements, global assessment, dietary questionnaire, and self-perception of health and nutrition^24^. The maximum global score for MNA-SF is 30 points, with the following classification: a normal nutritional status indicated by a score of 24 to 30, risk of malnutrition with scores between 17 and 23.5, and malnutrition indicated by a score less than 17 points^18^.

*Morisky Green Levine (MGL) adherence scale:* It has been validated across various chronic diseases to assess medication adherence in patients with HBP^25^. Since its introduction, the scale has been used to assess therapeutic adherence across different diseases due to its high specificity (0.92) and positive predictive value. The MGL scale consist of four questions with dichotomous answers (yes/no) that explore the patient's behavior regarding medication adherence. Its primary aim is to gauge whether patients exhibit appropriate attitudes toward their treatment regimen for their disease; incorrect attitudes are indicative of non-adherence. The patient is considered adherent when all questions are answered correctly. One of its advantages is the insight it provides into the reasons behind non- adherence^26^.

*Geriatric Depression Scale (GDS):* It is a widely used instrument for depression screening, translated and validated in various languages, including Spanish.

For this study, the five-item version of the GDS was used, which turned out to be as effective as the 15-question version, with a 97% sensitivity and 85% specificity for depression diagnosis. A score greater than two suggests depression, and less than two indicates the absence of depression^27^.

The data were analyzed using descriptive analysis on SPSS version 25 software. In addition, Chi-square and Fisher's exact tests were used to analyze categorical variables, while Wilcoxon-Mann-Whitney, Brunner-Munzel, Welch, and Student's t tests were used to analyze numerical variables. In order to select the variables for the final analysis, all variables were entered into a process called the Least Absolute Shrinkage and Selection Operator (LASSO), utilizing a 10-fold cross-validation method. This method involves randomly dividing the sample into ten equal parts. Subsequently, the first part is removed, and the model is fitted with the remaining nine parts to evaluate how well it can predict the information from the removed part. This process is repeated for all parts, and the error measured across the partitions is called a cross-validation error. Then, in multiple logistic regression, the variables are selected to minimize this cross-validation error with some tolerance, resulting in the final model containing only the selected variables. All statistical tests yielded a p-value less than 0.05.

The study was approved by the Ethics Committee of the School of Nursing of the Universidad Nacional de Trujillo, with reference number 003-CIEI-UNT. All participants provided signed informed consent forms and were provided with a copy.

## Results

Among the 342 participants of the study, 197 (57.60%) did not adhere to the HBP pharmacological treatment. A predominant trend of non-adherence to pharmacological treatment was observed among the participants across most of the sociodemographic variables studied. Adherence to treatment was identified among individuals with a university education (55.30%), retirees (50.40%), and those not living alone (57.50%). Furthermore, a relationship was found between retirement status and MGL adherence scale scores (Table 1).

**Table 1:** Relationship between adherence to hypertension pharmacological treatment and demographic and health variables among elderly residents of Trujillo City, 2018

Variable	Adherence (145) n (%)	Non-adherence (197) n (%)	p-value
Sex			0.77*
Female	92 (41.80)	128 (58.20)	
Male	53 (43.40)	69 (56.60)	
Marital status			0.28*
With a partner	80 (40.00)	120 (60.00)	
Without a partner	65 (45.80)	77 (54.20)	
Level of education			0.10*
No education	13 (27.10)	35 (72.90)	
Elementary school	68 (42.07)	91 (57.30)	
High school	43 (48.90)	45 (51.10)	
University education	26 (55.30)	21 (44.70)	
Retired			0.01*
Yes	69 (50.40)	68 (49.60)	
Not	76 (37.10)	129 (62.90)	
Live alone			0.84*
Yes	9 (60.00)	6 (40.00)	
Not	188 (57.50)	139 (42.50)	
Smoking			0.14**
Yes	0 (0.00)	4 (100.00)	
Not	145 (42.90)	193 (57.10)	
Drinks			0.35*
Yes	17 (36.20)	30 (63.80)	
Not	128 (43.40)	167(56.60)	

*Chi-square test; **Fisher's exact test

When means were compared, higher means were observed in variables such as systolic blood pressure, body mass index, calf circumference, cognitive status, depressive symptoms, and time of the disease, which exhibited non-adherence to HBP treatment. In addition, it was found that age (p=0.006), wage (p=0.03), and the presence of depressive symptoms (p=0.001) were associated with MGL scale scores (Table 2).

**Table 2:** Comparison of means between MGL adherence scale scores and the demographic and clinical variables among elderly residents of Trujillo City, 2018

Variable	Adherence (145) n (%)	Non-adherence (197) n (%)	p-value
Age	75.41 ± 8.23	72.95 ± 7.68	0.006*
Wage	536.36 ± 798.7	452.45 ± 1529.0	0.03**
Systolic Blood Pressure	125.11 ± 18.54	127.52 ± 17.43	0.17*
Diastolic Blood Pressure	76.03 ± 15.07	75.73 ± 13.60	0.61*
Weight	63.51 ± 11.52	63.45 ± 13.42	0.96 ||
Height	1.56 ± 0.09	1.55 ± 0.09	0.59 *⁋*
Body Mass Index	26.26 ± 5.15	26.30 ± 5.13	0.70**
Mid-upper arm circumference	27.21 ± 5.58	27.02 ± 5.12	0.55*
Calf circumference	32.43 ± 4.59	32.49 ± 4.79	0.32*
Cognitive status	16.28 ± 3.31	16.36 ± 2.40	0.36*
Nutritional condition	23.01 ± 5.88	22.57 ± 5.52	0.16*
Depressive symptoms	0.86 ± 1.25	1.20 ± 1.38	0.01*
Duration of hypertension	10.04 ± 7.95	10.12 ± 9.25	0.63*

*Wilcoxon Mann Whitney test; **Test Brunner Munzel; || Welch test; ⁋Student's t-test

Regarding self-reported diseases, it was observed that participants who reported having diabetes mellitus (63.50%) and Alzheimer's disease (100%) were non-adherent to treatment. Conversely, individuals with a history of stroke (85.70%) demonstrated significant adherence to treatment (p=0.001) (Table 3).

**Table 3:** Relationship between adherence to hypertension pharmacological treatment and self reported diseases among elderly residents of Trujillo City, 2018

Variable	Adherence n (%)	Non-adherence n (%)	p-value
Diabetes mellitus			0.35*
Yes	19 (36.50)	33 (63.50)	
Not	126 (43.40)	164 (56.60)	
Stroke			<0.001*
Yes	12 (85.70)	2 (14.30)	
Not	133 (40.50)	195 (59.50)	
COPD			0.75*
Yes	2 (50.00)	2 (50.00)	
Not	143 (42.30)	195 (57.70)	
Coronary heart disease			0.66*
Yes	4 (50.00)	4 (50.00)	
Not	141 (42.20)	193 (57.80)	
Cancer			0.70*
Yes	3 (50.00)	3 (50.00)	
Not	142 (42.30)	194 (57.70)	
Alzheimer’s disease			1.00**
Yes	0 (0.00)	1 (100.00)	
Not	145 (42.50)	196 (57.50)	

*Chi square test; **Fisher's exact test; COPD = Chronic Obstructive Pulmonary Disease

In the correlation analysis, it was identified that the factors that favor adherence to treatment were age (p=0.01), retirement status (p=0.05), and a history of stroke (p=0.004) (Table 4).

**Table 4:** Association between adherence to hypertension pharmacological treatment and demographic, health, and clinical variables and self-reported diseases among elderly residents of Trujillo City, 2018

Variables	OR	(CI 95%)	p-value
Age	1.03	(1.01 - 1.07)	0.01
Retired (not)	1.57	(1.00 - 2.47)	0.05
Stroke (not)	9.33	(2.43 - 61.40)	0.004

The variables excluded from the model were marital status, level of education, wage, living alone, smoking, drinking, systolic and diastolic blood pressure, weight, height, BMI, mid-upper arm and calf circumferences, cognitive status, nutritional status, depressive symptoms, duration of hypertension, diabetes mellitus, COPD, coronary heart disease, cancer, and Alzheimer's disease.

## Discussion

The study showed that more than half of hypertensive older adults did not adhere to drug treatment, and the factors that improve it are age, being retired, and having suffered a stroke.

In relation to the high prevalence found in the study, a systematic review of 13 studies from Western and non-Western countries conducted between 2000 and 2018 identified that non-adherence in Western countries was 16.13% and in non-Western, 45.7%^28^.

Non-adherence to treatment is a health problem that prevails in all age groups, but its incidence is higher in older adults^29^. Furthermore, it is associated with not only poor blood pressure control and increased hospital admission rate^30^ but also an acceleration in the development of serious complications and morbidities^31^, which can affect the quality of life.

Adhering to timely, regular, and long-term treatment could alleviate symptoms, control disease progression, prevent complications, and reduce mortality^28^^, ^^32^.

It was observed that the older the individuals, the better their adherence to hypertension treatment. However, various studies on the subject have yielded different results. For instance, some research suggests that the age group between 65 and 69 (26.6%) and 60 to 64 (23.1%) demonstrates notably higher adherence^33^^, ^^34^.

As individuals age, adherence to treatment often declines, accompanied by various vulnerabilities, such as decreased physical, mental, and cognitive abilities. This situation makes it difficult to understand and adhere to pharmacological and non-pharmacological treatments^34^, contrary to the results. Conversely, a review of 13 studies revealed an association between adherence and advanced age^28^.

Understanding this association is complex because older adults may experience greater comorbidity and perceive themselves as having poorer health. However, this adherence may be compromised by cognitive deficits, functional limitations, and self-care problems that should be considered during the evaluation process^35^^, ^^36^.

This study also found that retirement is associated with better adherence to pharmacological treatment for HBP. Previous studies have shown that employees were less likely to adhere to antihypertensive treatment, whereas retirees with hypertension exhibited higher adherence^37^^, ^^38^.

On the other hand, during the COVID-19 pandemic, a study was conducted in which half of the participants were classified as non-adherent to antihypertensive treatment. The primary contributing factor to non-adherence was forgetfulness (35.6%), followed by socioeconomic factors such as unemployment (75.8%) and retirement (70.9%)^39^.

Adherence to hypertension treatment is multidimensional, determined by various reciprocally interacting factors. These factors can be grouped into socioeconomic factors, those related to the healthcare team or system, those related to the characteristics of the disease itself, those related to the treatment, and patient-related factors^40^.

One model that would explain adherence to treatment in retirees is the Interaction Model of Client Health Behavior. This model is a socio-behavioral framework encompassing variables related to treatment, situational characteristics such as retirement, as well as subjective factors, including social, personal and economic consequences^41^.

Considering that hypertension is a health problem requiring long-term treatment, older adults dedicate years to adhering to their treatment regimen. Once a person has developed a propensity for healthy behavior, good intentions evolve into detailed instructions for carrying out desired actions. However, achieving this goes beyond wanting something; it requires cultivating self-regulatory skills and strategies. These include perceived self-efficacy in planning, taking action, and evaluating the results of one's actions^42^.

Non-adherence to treatment among older people with HBP is not only associated primarily with inadequate blood pressure control and more recurrent hospital stays^28^ but also hastens the onset of severe morbidities^31^ that can impact the quality of life.

The Precaution Adoption Model (PAPM) proposes health behavior change by encouraging favorable behaviors that replace harmful behaviors. This model suggests that progress through its stages requires understanding of the risks, costs, personal vulnerability, and benefits^43^.

In cases where the risk persists despite awareness of the danger, it may stem from factors such as lack of information, motivation issues, vulnerability, high perception of costs, and lack of self-determination to adopt a behavior^43^. These aspects could explain why the majority of older adults in the current study are non-adherent to hypertension treatment.

Another factor that improves adherence to drug treatment is having a history of stroke. An Australian study examined the adherence to antihypertensive treatment in the first six months after a stroke and found that older adults exhibited high adherence to antihypertensives, leading to a reduced risk of new episodes^44^.

This awareness serves as an additional motivation for them to follow the prescribed treatment, aiming to control blood pressure and minimize the chances of experiencing another stroke^44^^, ^^45^. By understanding the relationship between hypertension-specific complications and adherence to treatment, public health interventions can be designed to provide optimal care to this population and prevent the development of major complications associated with HBP^46^.

However, studies have shown an association between stroke and reduced adherence to drug therapy, as well as a decline in resuming social activities and overall quality of life. This situation can impact patient adherence to recommended self-care activities, including diet and exercise^47^^, ^^48^.Thisassociation was identified in a study conducted in Lebanon, in which stroke patients with functional disabilities as sequelae faced challenges in adhering to hypertension treatment^49^.

Health professionals collaborate to improve adherence to pharmacological treatment among hypertensive patients, particularly nurses who carry out educational activities. An intervention study demonstrated reduced blood pressure, increased medication adherence, and greater knowledge of the disease after six weeks of educational sessions^50^.

Furthermore, the use of technology has helped to improve adherence to pharmacological treatment. Monitoring over a 12-week period showed a beneficial effect in reducing blood pressure, as well as improving adherence to drug treatment^51^.

One of the study's limitations is its cross-sectional design, which does not allow making causal inferences. However, as the study includes a representative sample of the older adult population in Trujillo, statistical inferences can be drawn.

## Conclusion

This study showed that age, retirement status, and a history of stroke are variables favoring adherence to HBP treatment. Non-adherence to HBP treatment impedes the therapeutic goals. Therefore, this study underscores the need to establish or reestablish strategies that actively involve people with HBP and their caregivers to enhance their quality of life with the aim of minimizing or preventing this evident problem.

The findings suggest the necessity of implementing educational initiatives to strengthen self-care practices and provide a better understanding of the significance of identifying factors conducive to improved adherence to antihypertensive therapy. These measures are important to reduce damage and prevent complications derived from the disease. Moreover, it is imperative to highlight the pivotal role of nursing professionals in educating and caring for this population.

## References

[1] World Health Organization (2023). Decade of Healthy Ageing 2021-2030.

[2] Linares-Cánovas LP, Linares-Cánovas LB, Vitón-Castillo AA (2020). Determinación de la adherencia farmacológica en adultos mayores hipertensos. Aten Fam.

[3] Monterrey-Hernández M, Linares-Canovas L, Toledo-del-LLano R, Vazquez-Ramos A, Morales-Monterrey C (2021). Medication adherence and quality of life related to the health condition in hypertensive older adults. Rev cienc med Pinar Río.

[4] Pan American Health Organization (2019). Prevalence of hypertension among adults aged 30-79 years in countries of the Americas.

[5] González Rodríguez R, Lozano Cordero JG, Aguilar Méndez A, Gómez Domínguez OL, Díaz LM (2017). Caracterización de adultos mayores hipertensos en un área de salud. Rev cuba med gen integr.

[6] Brown MT, Bussell JK (2011). Medication adherence: who cares?. Mayo Clin Proc.

[7] Martín Alfonso LA, Grau Ábalo JA, Espinosa Brito AD (2014). Conceptual framework for evaluating and improving adherence to medical treatment in chronic diseases. Rev Cubana Salud Pública.

[8] Nielsen J0, Shrestha AD, Neupane D, Kallestrup P (2017). Non-adherence to anti-hypertensive medication in low- and middle-income countries: a systematic review and meta-analysis of 92443 subjects. J Hum Hypertens.

[9] Eschenhagen T (2018). Tratamiento de la hipertensión. In: Goodman, Gilman’s. The Pharmacological Basis of Therapeutics. Global Education Holdings.

[10] Pan J, Wu L, Wang H, Lei T, Hu B, Xue X (2019). Determinants of hypertension treatment adherence among a Chinese population using the therapeutic adherence scale for hypertensive patients. Medicine (Baltimore).

[11] Williams B, Mancia G, Spiering W, Agabiti Rosei E, Azizi M, Burnier M (2018). Guidelines for the management of arterial hypertension: The Task Force for the management of arterial hypertension of the European Society of Cardiology and the European Society of Hypertension. J Hypertens.

[12] Burnier M, Egan BM (2019). Adherence in Hypertension. Circ Res.

[13] Instituto Nacional de Estadística e Informática (2016). Situación de salud de la Población Adulta mayor.

[14] Fhon JRS, Gómez-Luján MP, Caetano GM, Cáceda Nazco GS, Dos Santos NAP, Leitón-Espinoza ZE (2024). Factors associated with adherence to hypertensive pharmacological treatment in the older adult. Mendeley Data V1.

[15] Kish L (1949). A procedure for objective respondent selection within the household. Journal of the American Statistical Association.

[16] Center for disease control and prevention (2017). How to measure height and weight accurately at home.

[17] Ministerio de salud (2012). Guía Técnica para la Valoración Nutricional Antropométrica de la Persona Adulta. Instituto Nacional de salud del Perú.

[18] Guigoz Y, Vellas B, Garry PJ (1996). Assessing the Nutritional Status of the Elderly: the Mini Nutritional Assessment as Part of the Geriatric Evaluation. Nutr Rev.

[19] Serra RJA, Cuesta TF, García LMA, Ruipérez CI (2007). Valoración nutricional en el anciano: recomendaciones prácticas de los expertos en geriatría y nutrición.

[20] World Health Organization (2006). Manual de vigilancia STEPS de la OMS: el método STEPwise de la OMS para la vigilancia de los factores de riesgo de las enfermedades crónicas.

[21] Chobanian AV, Bakris GL, Black HR, Cushman WC, Green LA, Izzo JL (2003). Seventh report of the Joint National Committee on Prevention, Detection, Evaluation, and Treatment of High Blood Pressure. Hypertension.

[22] Laguna JY, Arbeloa GL (2010). Manual de Descripción de los Grupos Relacionados por el Diagnóstico. (AP-GRD v 25.0).

[23] Icaza MG, Albala C (1999). Minimental State Examinations (MMSE) del estudio de la demencia en Chile: Análisis Estadístico. Serie Investigaciones en Salud Pública.

[24] Guigoz Y, Vellas B, Garry PJ, Vellas B (2004). The Mini Nutritional Assessment (MNA).

[25] Morisky DE, Green LW, Levine DM (1986). Validez concurrente y predictiva de una medida autoinformada de adherencia a la medicación. Med Care.

[26] Ortega L (2004). Uso de los Cuestionarios para medir la Adherencia al tratamiento Antirretroviral.

[27] Hoyl T, Valenzuela E, Marín PP (2000). Depresión en el adulto mayor: evaluación preliminar de la efectividad, como instrumento de tamizaje, de la versión de 5 ítems de la Escala de Depresión Geriátrica. Rev. méd. Chile.

[28] Berlowitz DR, Foy CG, Kazis LE, Bolin LP, Conroy MB, Fizpatrick P, Gure TR. (2017). Effect of intensive blood-pressure treatment on patient-reported outcomes. N. Engl. J. Med.

[29] Khayyat SM, Khayyat SMS, Hyat ARS, Mohamed MMA, Abdul HM (2017). Predictors of medication adherence and blood pressure control among Saudi hypertensive patients attending primary care clinics: a cross-sectional study. PLoS One.

[30] Uchmanowicz B, Jankowska EA, Uchmanowicz I, Morisky DE (2019). Self-reported medication adherence measured with morisky medication adherence scales and its determinants in hypertensive patients aged >60 years: A systematic review and meta-analysis. Front pharmacol.

[31] Wang W, Luan W, Zhang Z, Mei Y (2023). Association between medication literacy and medication adherence and the mediating effect of self-efficacy in older people with multimorbidity. BMC geriatrics.

[32] Xue J, Conwell Y, Tang W, Bogner HR, Li Y, Jiang Y (2019). Treatment adherence as a mediator of blood pressure control in Chinese older adults with depression. Int J Geriatr Psychiatry.

[33] Shi S, Shen Z, Duan Y, Ding S, Zhong Z (2019). Association between medication literacy and medication adherence among patients with hypertension. Front Pharmacol.

[34] Yang C, Hui Z, Zeng D, Zhu S, Wang X, Lee DTF (2021). A community-based nurse-led medication self-management intervention in the improvement of medication adherence in older patients with multimorbidity: protocol for a randomized controlled trial. BMC Geriatr.

[35] Burgal-Cintra CJ, Pérez-Bichor A, Ortega-López IL (2021). Caracterización de la adherencia terapéutica en adultos mayores hipertensos. Rev Méd Electrón.

[36] Gewehr DM, Bandeira VAC, Gelatti GT, Colet CF, Oliveira KR (2018). Adherence to pharmacological treatment of arterial hypertension in Primary Health Care. Saúde debate.

[37] Kang CD, Tsang PPM, Li WTL, Wang HHX, Liu KQL, Griffiths SM (2015). Determinants of medication adherence and blood pressure control among hypertensive patients in Hong Kong: a cross-sectional study. Int. J. Cardiol.

[38] Li YT, Wang HHX, Liu KQL, Lee GKY, Chan WM, Griffiths SM (2016). Medication adherence and blood pressure control among hypertensive patients with coexisting long-term conditions in primary care settings: a cross-sectional analysis. Medicine (Baltimore).

[39] Guimaraes MCLP, Coelho JC, Santos J, Higa CBO, Florido CF, Lee RJW (2022). Adherence to antihypertensive treatment during the COVID-19 pandemic: findings from a cross-sectional study. Clin Hypertens.

[40] Ramos Morales LE (2015). La adherencia al tratamiento en las enfermedades crónicas. Revista Cubana de Angiología y Cirugía Vascular.

[41] Heiby EM, Carlson JG (1986). The health compliance model. JCHC.

[42] Heiby EM, Lukens CL, Frank MR (2005). The Health Compliance Model II. The Behavior Analyst Today.

[43] Weinstein ND, Lyon JE, Sandman PM, Cuite CL (1998). Experimental evidence for stages of health behavior change: the precaution adoption process model applied to home radon testing. Health psychol.

[44] Dalli LL, Olaiya MT, Kim J, Andrew NE, Cadilhac DA, Ung D (2023). Antihypertensive medication adherence and the risk of vascular events and falls after stroke: a real-world effectiveness study using linked registry data. Hypertension.

[45] Gynnild MN, Aaker0y R, Spigset O, Askim T, Beyer MK, Ihle-Hansen H (2021). Vascular risk factor control and adherence to secondary preventive medication after ischemic stroke. J Intern Med.

[46] Lima DBS, Moreira TMM, Borges JXP, Rodrigues MTP (2016). Association between treatment compliance and different types of cardiovascular complications in arterial hypertension. Texto y Contexto Enfermagem.

[47] Shi Y, Yang D, Zeng Y, Wu W (2017). Risk factors for post-stroke depression: a meta-analysis. Front. Aging Neurosci.

[48] Arowoiya AI, Elloker T, Karachi F, Mlenzana N, Khuabi LJ, Rhoda A (2017). Using the World Health Organization's disability assessment schedule (2) to assess disability in community-dwelling stroke patients. Afr. J. Physiother.

[49] Sakr F, Dabbous M, Akel M, Salameh P, Hosseini H (2022). Adherence to post-stroke pharmacotherapy: scale validation and correlates among a sample of stroke survivors. Medicina (Kaunas).

[50] Marseille BR Commodore-MensahY, Davidson PM Baker D, D’Aoust R Baptiste DL (2021). Improving hypertension knowledge, medication adherence, and blood pressure control: a feasibility study. J Clin Nur.

[51] Kes D, Polat U (2022). The effect of nurse-led telephone support on adherence to blood pressure control and drug treatment in individuals with primary hypertension: A randomized controlled study. Int J Nurs Pract.

